# Editorial: Many-Body Green’s Functions and the Bethe-Salpeter Equation in Chemistry: From Single Molecules to Complex Systems

**DOI:** 10.3389/fchem.2022.866492

**Published:** 2022-02-21

**Authors:** Marc Dvorak, Björn Baumeier, Dorothea Golze, Linn Leppert, Patrick Rinke

**Affiliations:** ^1^ Department of Applied Physics, Aalto University, Espoo, Finland; ^2^ Department of Mathematics and Computer Science and Institute for Complex Molecular Systems, Eindhoven University of Technology, Eindhoven, Netherlands; ^3^ Faculty of Chemistry and Food Chemistry, Technische Universität Dresden, Dresden, Germany; ^4^ MESA+ Institute for Nanotechnology, University of Twente, Enschede, Netherlands

**Keywords:** many-body Green’s function, GW approximation, Bethe-Salpeter equation, computational chemistry, theoretical spectroscopy

The utility of many-body Green’s function methods for computing excitation energies and spectra of correlated systems continues to impress. While applications and implementations of the *GW* approach were initially developed in the solid-state physics community, the method and its combination with the Bethe-Salpeter Equation (BSE) has more recently taken a foothold among quantum chemists. Across disciplines, the field continues to grow and it currently carries great momentum: it is therefore timely to collect articles showcasing the latest triumphs and outstanding shortcomings of this family of methods.

The contributions to this Research Topic range from theory and method development to applications of Green’s function-based methods. Applications of *GW* to organic crystals and FeS_2_ are motivated by the importance of an atomistic understanding of excited states for technological applications such as photovoltaics. Contributions that highlight the predictive power of the *GW* approach and present low-scaling implementations showcase the potential of the theory for applications to complex heterogeneous materials. Additionally, method and theory development for *GW*, BSE, and non-*GW* calculations of the single-particle Green’s function form important parts of this collection of articles.

In *The GW/BSE Method in Magnetic Fields*, Holzer et al. benchmark their new *GW*/BSE implementation in external magnetic fields. External fields pose a challenge to electronic structure theory because they require complex orbitals to ensure gauge-invariant results, raising the computational expense. Furthermore, kernels of time-dependent density functional theory can be unstable in the presence of the perturbing field. *GW*/BSE presents a solution to the latter problem, and the authors implement the theory with complex London orbitals to circumvent gauge dependence.

The fundamental band gaps of two phases of FeS_2_ remain somewhat undetermined: theoretical calculations are scattered and experiments only reliably measure the optical band gap. In *Accurate Prediction of Band Structure of* FeS_2_ *: A Hard Quest of Advanced First-Principles Approaches*, Zhang and Jiang set out to resolve the theoretical discrepancy by performing *GW* calculations on both phases with the most complete basis to date: a linearized augmented plane wave basis (LAPW) supplemented with high-energy local orbitals (HLOs). The authors find that very high momentum HLOs are necessary to converge the sum over unoccupied states in *GW* and attribute inconsistencies in previous theoretical work to underconvergence.

Molecular crystals are a modern success of *GW*. Their high tunability through packing and choice of substrate render them an interesting playing field for computational exploration. The *GW* approach affords an excellent description of the important effect of substrate screening. In *Exciton Modulation in Perylene-Based Molecular Crystals Upon Formation of a Metal-Organic Interface From Many-Body Perturbation Theory*, Shunak et al. explore the change in quasiparticle and excitonic properties of octyl perylene diimide (C8-PDI) adsorbed on gold. They find that exciton binding energies are unchanged due to the local character of screening between electron and hole but that fundamental band gaps are dependent on layer number, stacking, and substrate.

Most low-scaling *GW* algorithms are restricted to diagonal *G*_0_ *W*_0_. In *Low-Order Scaling Quasiparticle Self-Consistent GW for Molecules*, Förster and Visscher present their low-scaling implementation of quasiparticle self-consistent *GW* (qsGW) based on the space-time method. They validate their implementation against other results and then include a polarizable continuum model to study DNA oligomers.

The cumulant approximation makes an exponential ansatz to the Green’s function $G_{c}\left(t\right)=G_{c}^{0}\left(t\right)e^{C\left(t\right)}$ , and is known to produce satellite and shake-up features of the spectral function better than Dyson-equation-based calculations. *Equation-of-Motion Coupled-Cluster Cumulant Green’s Function for Excited States and X-Ray Spectra* by Vila et al. presents a real-time formulation for computing X-ray spectra that draws on connections between coupled-cluster and cumulant approaches. Core ionization energies from the improved EOM-CC method are presented for CH_4_, NH_3_, H_2_O, HF, and Ne, and a full X-ray absorption spectrum is presented for NH_3_.

The Hubbard model remains a valuable test bed for theoretical physics since it permits both analytic and numerically exact solutions in certain cases. *Scrutinizing GW-Based Methods Using the Hubbard Dimer* by Di Sabatino et al. finds that different starting points for constructing *G*_0_ and *W*_0_ lead to the existence of multiple quasiparticle solutions, loss of particle number, or discontinuities in physical quantitites as a function of interaction strength. Fully self-consistent *GW* removes these artifacts but does not necessarily improve the accuracy of the results.

*The GW Miracle in Many-Body Perturbation Theory for the Ionization Potential of Molecules* systematically tests ionization potentials for the *GW*100 test set computed with *G*_0_ *W*_0_ against a series of alternative many-body theories. Bruneval et al. find that, while more accurate theories may exist, they come at much greater expense; the cost-accuracy balance of *G*_0_ *W*_0_ is unbeatable. They also note that the most substantial improvement to *G*_0_ *W*_0_ comes not by incorporating additional diagrams but from an improved mean-field starting point.

Calculations of optical spectra in solids with the Bethe-Salpeter equation require an enormously dense **k**-point grid due to the extended character of excitons in real space. This remains a bottleneck in applying the method to solids. *Double k-Grid Method for Solving the Bethe-Salpeter Equation via Lanczos Approaches* by Alliati et al. presents an efficient interpolation scheme to ameliorate this problem. It computes the optical absorption on a fine **k**-grid, on which each diagonal block of the Hamiltonian is taken from the calculation of the kernel on a coarse **k**-grid. The scheme reproduces optical spectra of Si, GaAs, and MoS_2_ with good accuracy.

In *Photoemission Spectra from the Extended Koopman’s Theorem, Revisited*, Di Sabatino et al. show that their recent work developing many-body effective energy theory (MEET) is more closely related to the well-known Extended Koopman’s Theorem (EKT) than previously appreciated. The lowest level of approximation to MEET is equivalent to the diagonal approximation of the EKT. The authors explore the asymmetric Hubbard dimer and find that the MEET energies depend on the choice of basis, corroborating previous work.

The continued development of theory and algorithms, progression of computational power, and importance of excited state spectra ensure that many-body Green’s functions will play a role in fundamental science for the forseeable future.

